# Associations of ^13^C-Sucrose Breath Test Dynamics with Anthropometry and Demographics: A Comparison of Studies in the United Kingdom and Zambia

**DOI:** 10.1016/j.cdnut.2025.107590

**Published:** 2025-10-30

**Authors:** Stephanie P Iorga, Hannah Van Wyk, Gwenyth O Lee, Robert J Schillinger, Christine A Edwards, Phoebe Hodges, Ellen Besa, Paul Kelly, Douglas J Morrison, Andrew F Brouwer

**Affiliations:** 1 Department of Epidemiology, University of Michigan, Ann Arbor, MI, United States; 2 Rutgers Global Health Institute, Rutgers University, New Brunswick, NJ, United States; 3 Scottish Universities Environmental Research Centre, University of Glasgow, East Kilbride, United Kingdom; 4 School of Medicine, Dentistry and Nursing, University of Glasgow, Glasgow, United Kingdom; 5 Tropical Gastroenterology and Nutrition group, University of Zambia School of Medicine, Lusaka, Zambia; 6 Blizard Institute, Barts and The London School of Medicine, London, United Kingdom

**Keywords:** ^13^C, sucrose breath test, environmental enteric dysfunction, anthropometry, mechanistic model

## Abstract

**Background:**

The ^13^C-sucrose breath test (^13^C-SBT) is a noninvasive diagnostic test that has been used to assess intestinal sucrase-isomaltase activity, which may be altered in gut function disorders such as environmental enteric dysfunction (EED), a syndrome characterized by chronic inflammation and histologic changes in the small intestine and thought to be ubiquitous among people living without access to improved water and sanitation. However, characterizing associations between ^13^C-SBT breath curves and gut function status requires disaggregating associations with sucrase-isomaltase activity from associations with other aspects of sucrose metabolism.

**Objectives:**

This analysis aimed to identify anthropometric and demographic patterns in ^13^C-SBT breath curves.

**Methods:**

We investigated the associations between anthropometry (height, weight, body mass index) or demographics (sex, age) and each of 3 mechanistic pharmacokinetic model parameters estimated from ^13^C-SBT breath curves, comparing adult populations in United Kingdom and Zambia, who have different risk of EED.

**Results:**

Zambian participants had higher values of the parameter associated with intestinal sucrase-isomaltase activity and liver metabolism [ρ: 2.4 (United Kingdom) compared with 3.5 (Zambia), *P =* 0.005] and higher fractions of tracer recovery [κ: 0.69 (United Kingdom) compared with 0.93 (Zambia), *P* <0.001]. The rate-limiting parameter, speculated to be associated with pulmonary excretion of plasma bicarbonate as CO_2_, was lower among Zambian participants [πρ: 0.30 (United Kingdom) compared with 0.22 (Zambia), *P =* 0.009]. We found a similar association between the rate-limiting parameter (πρ) and weight in both cohorts, with higher weight associated with slower tracer recovery. We did not find significant associations between anthropometry and the parameter associated with sucrase-isomaltase activity (ρ), helping to alleviate concerns about potential confounding by anthropometry when using ρ as a model-based ^13^C-SBT diagnostic of intestinal sucrase-isomaltase activity.

**Conclusions:**

The associations we identified between weight and the rate-limiting parameter (πρ) should be further investigated and better understood mechanistically. These anthropometric associations identified for adults should be further confirmed in infants and children.

## Introduction

Environmental enteric dysfunction (EED), an acquired gut function disorder affecting both adults and children, is characterized by gut inflammation, small-intestinal villous atrophy, and a diminished crypt-to-villus ratio [1]. EED is associated with adverse health outcomes, notably chronic malnutrition, among children living in socioeconomically disadvantaged settings [1,2]. Factors such as prolonged exposure to unsanitary environmental conditions, early-life exposures to pathogens, malnutrition, and gut microbiome dysbiosis may all contribute to the development and progression of EED [3]. However, understanding EED, its risk factors, and outcomes has been challenging, as EED lacks a universally accepted case definition or specific diagnostic criteria and often persists without clinical symptoms [3], making it challenging to diagnose noninvasively. The reference-standard diagnostic approach for identifying EED has relied on histological examination of biopsies of the small intestine to identify villous blunting, crypt hypertrophy, diminished villous height to crypt depth ratio, and epithelial breakdown [4,5]. Although effective, biopsies are costly, time consuming, and require highly trained medical personnel, posing a challenge for resource-limited populations, and, because EED can present without clear symptoms, performing invasive procedures in asymptomatic individuals raises ethical concerns.

Our group has developed a stable isotope ^13^C-sucrose breath test (^13^C-SBT) as a potential noninvasive and cost-effective alternative for detecting EED [6]. This test relies on the detection of ^13^CO_2_ in exhaled breath after the oral ingestion of ^13^C-labeled sucrose, which is metabolized in the gut brush-border primarily into glucose and fructose by the enzyme sucrase-isomaltase, a glucosidase enzyme most abundantly expressed at the villous tips of the gut epithelium. These monosaccharides are then transported across the gut and metabolized, yielding ^13^C-labeled CO_2_ that can be measured in breath samples. Because EED is associated with villous blunting, we theorized that sucrase-isomaltase activity may be diminished in people with EED. Previous work has suggested that the ^13^C-SBT may be able to detect EED in children [7], and our own previous work has shown that induced inhibition of sucrase in adults can be detected by the ^13^C-SBT [8]. Breath tests using ^13^C-sucrose and other ^13^C substrates have been proposed to investigate a variety of gut disorders, including congenital sucrase-isomaltase deficiency [9,10], celiac disease [11], functional bowel disorders [12], and mucositis induced by cytotoxic chemotherapy [13,14].

For the ^13^C-SBT to move from the realm of research to that of clinical utility, there needs to be a better understanding of the factors contributing to the variability in ^13^C-SBT outcomes across diverse populations, as well as their relationship to the pathophysiology of EED and other gut function disorders. For example, the underlying mechanisms driving the associations between ^13^C-SBT dynamics and anthropometry are not fully understood, and little is currently known about how ^13^C-SBT dynamics vary between populations in different geographical, diet, and water, sanitation, and hygiene contexts. There are concerns that ^13^C-SBT outcomes may be confounded by metabolic factors unrelated to sucrase-isomaltase activity. For example, calculation of ^13^C-SBT breath curves from isotopic measurements requires an anthropometry-based assumption of the rate of CO_2_ production ($V_{{CO}_{2}}$), which could be systematically biased for some populations.

Our exploratory study aims to investigate the associations between ^13^C-SBT breath curve dynamics and anthropometric variables such as height, weight, and BMI (in kg/m^2^), comparing an adult population in Glasgow, United Kingdom, where EED can be assumed to be absent, and a low socioeconomic status community in Lusaka, Zambia, where EED is thought to be highly prevalent. Using robust statistical analyses and mathematical modeling developed for the ^13^C-SBT [15], our work offers insights into how individual anthropometry may influence ^13^C-SBT breath curves across different populations. Our work will contribute to an enhanced understanding of the applicability of the ^13^C-SBT to EED and other gut function research and testing, and ultimately to the development of more targeted interventions for addressing the impact of EED and other gut function disorders on global health.

## Methods

### Overview

This analysis uses data from 2 ^13^C-SBT experiments performed in adults in Glasgow, United Kingdom, and Misisi, a low socioeconomic status settlement in Lusaka, Zambia. These 2 studies were part of the optimization and validation steps for a larger, coordinated research project [6], and this secondary modeling analysis should be viewed as exploratory, hypothesis generating, and intended to establish research protocols and expected results for larger, confirmatory studies. In brief, in both studies, participants ingested a highly enriched ^13^C-labeled sucrose tracer immediately prior to providing longitudinal breath samples at 15 min intervals, which were used to generate percent dose recovery rate (PDRr) curves over time. Although there were site-specific differences in testing protocols, prior work has established that these differences are unlikely to make a functional difference to the breath test dynamics. The doses of highly enriched sucrose administered in these studies are negligible relative to typical dietary sucrose intake, so any saturation effects are a result of intestinal function disorder and not a result of the dose itself. In previous protocol-establishing work [16], we demonstrated only small saturating effects in early dynamics when a 50 mg tracer dose was accompanied by “flooding dose” of 20 g unlabeled sucrose, a difference several orders of magnitude greater than the differences in dose between the studies. Additionally, although the full 3-d low ^13^C diet is a best practice when working with naturally enriched tracers, it is not needed in practice when working with the highly enriched tracers used in these studies [16].

Breath test curves in both studies were fit to a previously developed mechanistic model of the pharmacokinetics of sucrose transport and metabolism [8,15,17]. We then estimated associations between each of 3 mechanistic parameters, described below, with anthropometric measures in both locations stratified by location and combined. The mechanistic model supports inferences about the physiological drivers of associations between anthropometry and sucrase-isomaltase activity and other metabolic factors captured by the ^13^C-SBT.

### Data

For data collected from Glasgow, United Kingdom (data collected in 2018), adult participants were recruited by advertisement. This study included 19 adult participants who reported no symptoms or history of gastrointestinal disease. Each participant had to follow a strict, low ^13^C diet for 3 d and fast for 8 h prior to testing. Prior to data collection, each participant provided a baseline breath sample. Then, participants ingested a 50 mg dose of highly enriched ^13^C sucrose tracer (U-^13^C sucrose, ≥99 atom% ^13^C, Sigma Aldrich) dissolved in water, and provided breath samples in 12 ml breath sampling vials every 15 min for ≤8 h. The study was approved by the University of Glasgow College of Medical, Veterinary and Life Sciences Research Ethics Committee (Application Number: 200190155). All Participants provided written informed consent. This study was performed in accordance with the Declaration of Helsinki.

In Lusaka, Zambia (data collected in 2019), the study was advertised by word of mouth, and adults who wanted to participate were screened by interview and physical examination. Participants with clinical illnesses such as hypertension and cardiac murmur were excluded. Each of 24 participants underwent an overnight fast. On the day of data collection, each participant provided a baseline breath sample and then ingested a 0.3 mg/kg body weight dose (∼20 mg at mean body weight) of highly enriched ^13^C sucrose tracer (as above) in water and provided breath samples every 15 min for ≤4 h. After sucrose breath testing, each participant from Zambia underwent a small-intestinal biopsy, recording measurements such as villous height and crypt depth [16]. We excluded data from 2 participants whose breath tests were cut short (<2 h), as recommended by our previous analysis [17]. The study was approved by the Excellence in Research Ethics and Science Converge Institutional Review Board (10 December, 2018). Participants provided written informed consent. This study was performed in accordance with the Declaration of Helsinki.

In both studies, breath tests were analyzed using isotope ratio mass spectrometry (IRMS AP-2003) using published protocols [18], and the results were converted to PDRr as previously described [15]. Demographic and anthropometric data, including participant age, sex, height, weight, and BMI, were recorded for each participant in both studies.

### Mechanistic model

**FIGURE 1:**
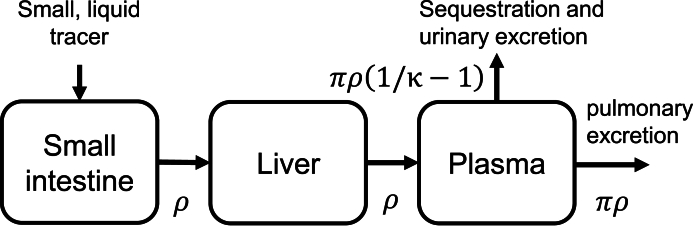
Pharmacokinetic model of the ^13^C-sucrose breath test. A structurally identifiable reduced pharmacokinetic model of physical transport, tracer metabolism, bicarbonate kinetics, and excretion in the ^13^C-sucrose breath test. This reduced model describes observed breath curves as the product of a γ-distributed process with mean rate ρ/2 and an exponentially distributed process (with mean rate πρ, where 0< π ≤ 1). Mathematically, we do not know which of these process is which biological process, but previous work suggests that small-intestinal and liver metabolism are associated with ρ [8], and we speculate that πρ is associated with pulmonary excretion from plasma bicarbonate. Parameter *κ* is the fraction of tracer that is excreted on the breath instead of through urine or being sequestered (κ=πρ/(πρ+πρ(1/κ−1)).

Our previously developed [8,15,17], mechanistic 3-parameter model of the ^13^C-SBT describes transport and metabolic dynamics underlying the breath test curve as a combination of a γ-distributed process (with rate parameter ρ and shape parameter 2) and an exponentially distributed process (with rate parameter πρ, where 0< π ≤ 1), along with the fraction of the tracer that is exhaled instead of stored or excreted through urine (κ). In previous work, we determined that the γ-distributed process captures both sucrose hydrolysis by sucrase-isomaltase in the small intestine and metabolism of glucose and fructose in the liver [8]. It is currently unknown which process(es) the exponentially distributed process accounts for, but we speculate that it is related to plasma bicarbonate kinetics. The model is illustrated in Figure 1. We fit the model to the data by minimizing the normal negative log likelihood as a function of the model parameters to obtain the maximum likelihood estimate of ρ, πρ, and κ, as previously described [8,15,17]. We then plot the fitted model curve over each participant's PDRr curve using the optimal parameter values from each participant.

We noted 1 participant from the Zambia study exhibited an unusual breath curve (an unusually steep decline in the breath curve), substantially different from all other participants in either country, causing us to label them as an outlier (Participant Zambia 9). We speculate that this participant had an unknown or undisclosed health condition. This participant was included in the scatterplots for completeness and can be identified by the star symbol, but they were excluded from all models and comparisons described below so that the results are representative of adults with typical breath curves. We emphasize that this current analysis is exploratory and preliminary, and any associations and potential outliers should be interrogated further in future work.

### Analysis

We estimated associations between the values of the mechanistic parameters ρ, πρ, and κ, and each anthropometric variable in linear regression models. To evaluate whether associations varied between study population, we first stratified by study, then combined both studies in models with a covariate × country interaction term. Because κ is a parameter bounded between 0 and 1, we used logit(κ) as the outcome variable for these models, effectively estimating the association for a logistic rather than a linear model. Additionally, we assessed whether weight was a potential confounder of the association between the other predictors and the mechanistic parameters through an exposure–covariate table.

Because anthropometry (height, weight, BMI) was strongly associated with age, sex, and study site, we then included all predictors and predictor × country interactions in an elastic net regression (ENET) to address multicollinearity and potential confounding, prevent overfitting, and aid in model interpretation [19]. ENET, which combines lasso and ridge regression techniques, allows the model to hone in on the most relevant predictors by shrinking the associations for less influential ones to zero [19]. In our analysis, the predictors were standardized to allow for easier comparison and interpretability in the regression model. In ENET, $\lambda_{1}$ and $\lambda_{2}$ are regularization parameters that control the extent of shrinkage applied to the coefficients of the predictors in a model. The larger the λ values, the greater the shrinkage, reducing coefficients toward zero. In this analysis, $\lambda_{1}$ and $\lambda_{2}$ were selected using 5-fold cross validation. All analyses were performed in R (v4.2.3; R Foundation for Statistical Computing) for each model parameter and each demographic and anthropometric variable.

## Results

### Anthropometry

The analysis included 41 total participants, 19 from the United Kingdom and 22 from Zambia. Anthropometry was collected from each participant, with no missing data (Table 1). The average age for United Kingdom participants was 22.9 y (range: 18–34 y) compared with 41.0 y (range: 17–59 y) for Zambian participants. The proportion of participants by sex was similar by site, with females representing 45.5% of the Zambia participants and 52.6% of the United Kingdom participants. The Zambian participants had on average higher BMI (25.7) compared with the United Kingdom participants (22.1), lower height (164 cm) compared with the United Kingdom (172 cm), and similar weight (67.6 kg) compared with the United Kingdom (65.6 kg).

**TABLE 1:** Descriptive characteristics of study participants by country.

Characteristics	Overall count (%) or mean (SD)	
	United Kingdom (*N* = 19)	Zambia (*N* = 22)
Sex		
Male	9 (47%)	12 (55%)
Female	10 (53%)	10 (45%)
Age (y)		
Mean (SD)	22.9 (4.42)	41.0 (13.8)
Median (min, max)	21 (18, 34)	45 (17, 59)
Weight (kg)	65.6 (14.5)	67.6 (16.4)
Height (cm)	172 (8.01)	164 (9.13)
BMI	22.1 (3.61)	25.7 (7.74)

### ^13^C-SBT test curves

The fits of the mathematical model to each participant’s breath test curve, represented by PDRr over time, are included in Supplemental Figures 1 (Zambia) and **2** (United Kingdom). For most participants, breath test curves show a unimodal curve with a steep incline on sucrose tracer ingestion, followed by a peak around the 2-h mark and a decline. The model captures the breath curve dynamics well, qualitatively.

### Associations between anthropometry or demographics and model parameters

**FIGURE 2:**
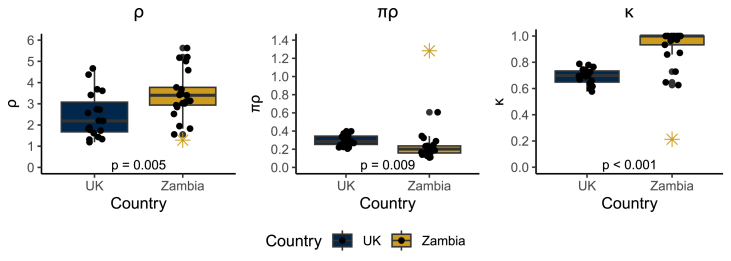
Mechanistic model parameter estimates (ρ, πρ, and κ) by country. Previous work suggests that small-intestinal and liver metabolism are associated with ρ, and we speculate that πρ is associated with pulmonary excretion from plasma bicarbonate. Parameter *κ* is the fraction of tracer that is excreted on the breath instead of through urine or being sequestered. *P* values for the difference in mean parameter estimates by country were estimated by linear regression. One participant with outlier breath test values is indicated by a star. Data collected in Glasgow, United Kingdom ( 2018) and in the Misisi settlement of Lusaka, Zambia (2019).

To investigate the associations between ^13^C-SBT parameters and anthropometry (height, weight, BMI) and demographics (sex, age), we plotted each model parameter against each predictor, stratifying by country. This approach allowed us to assess trends within groups and between groups. First, we evaluated each model parameter by country (Figure 2).

**FIGURE 3:**
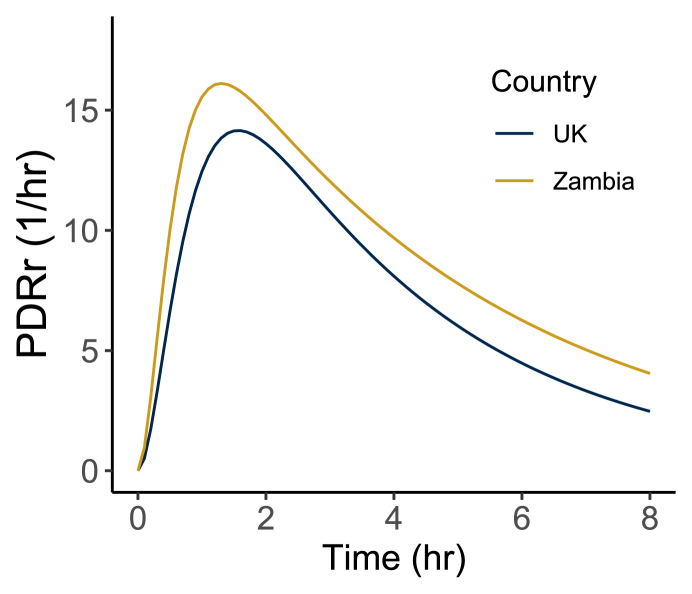
Modeled breath curves at the mean parameter values by country. PDRr, percent dose recovery rate.

There was a statistically significant difference in each parameter distribution between United Kingdom and Zambian participants [ρ: 2.4 (United Kingdom) compared with 3.5 (Zambia), *P =* 0.005; πρ: 0.30 (United Kingdom) compared with 0.22 (Zambia), *P =* 0.009, κ: 0.69 (United Kingdom) compared with 0.93 (Zambia), *P* <0.001]. On average, πρ was lower in Zambian participants compared with those in the United Kingdom, whereas ρ and κ were on average greater in Zambian participants compared with United Kingdom participants. Many Zambian participants were estimated to have values of κ near 1 (57% had κ > 0.99), indicating that virtually all of the tracer administered would be recovered on the breath, given a sufficiently long test duration. To visualize how these differences in parameters translated into differences in breath curves, we plotted the curves at the mean parameter values (not the same as the mean curves) in Figure 3. The higher value of ρ in Zambia compared with the United Kingdom can be seen in the faster initial increase in the PDRr, the lower value of πρ can be (subtly) seen in the slower decay rate after the peak, and the higher value of κ can be seen in the greater area under the curve.

**FIGURE 4:**
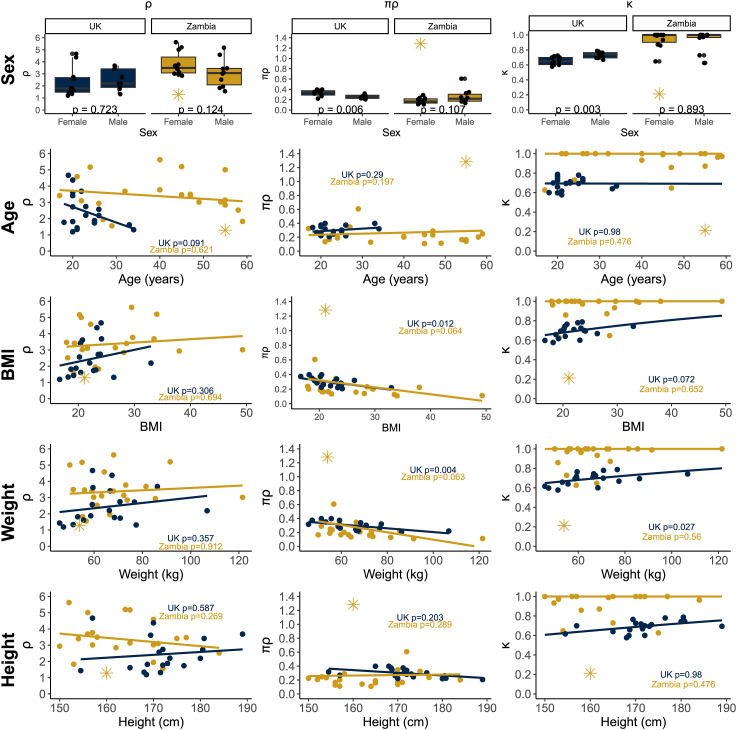
Associations between anthropometric variables and mechanistic breath curve parameters by country. Boxplot and scatterplots represent model parameter estimates for ρ, πρ, and κ (points) overlaid by the model line of best fit (line) for each participant. The outlier Zambian participant is represented by a yellow star symbol. The lines of best fit overlaying κ plots are the lines of best fit produced by a model using logit(κ) transformed values, and then untransformed using an expit function to produce more interpretable lines of best fit. The boxplots representing parameter estimates vs. sex indicate the *P* value for a 2-sample *t*-test; scatterplots indicate the *P* value for the Wald test for significance of the predictor. Data collected in Glasgow, United Kingdom (2018) and in Lusaka, Zambia (2019).

Figure 4 provides visual representation of participant anthropometric associations with each breath curve parameter by country. Unadjusted model estimates and corresponding *P* values are given in Table 2, and models accounting for effect modification by country are given in Table 3. Parameter ρ was only statistically significantly different between countries [2.4 (United Kingdom) compared with 3.5 (Zambia), *P =* 0.005], and logit(κ) had a statistically significant association with country (*P* < 0.001), and age (0.171/y, *P =* 0.027), although the association with age overall was driven by differences in the distribution of ages within country (Figure 3). Parameter πρ was statistically significantly associated with multiple parameters, most notably weight (−0.003 per kg; *P =* 0.002), BMI (−0.008 per unit, *P* < 0.001), and age (−0.003/y, *P =* 0.008). We assessed whether weight and age were potential confounders of the association between πρ and the other predictors (Supplemental Tables **1** and **2**). We found that weight was associated with height and sex in the United Kingdom cohort, with males making up the entirety of the highest quintile of weight. In the Zambian cohort, weight was associated with sex, where females made up the entirety of the fourth and fifth quintiles, but not height.

**TABLE 2:** Unadjusted regression models for associations between each mechanistic model parameters and each anthropometric and demographic predictor.

Parameter	Predictors	Coefficient	*P* value
ρ	Country (Glasgow vs. Zambia)	1.029	0.005^1^
	BMI (per unit)	0.045	0.135
	Weight (kg)	0.011	0.402
	Height (cm)	−0.032	0.111
	Age (y)	0.015	0.287
	Sex (female vs. male)	−0.317	0.411
πρ	Country (Glasgow vs. Zambia)	−0.079	0.009^1^
	BMI (per unit)	−0.008	<0.001^1^
	Weight (kg)	−0.003	0.002^1^
	Height (cm)	0.002	0.247
	Age (y)	−0.003	0.008
	Sex (female vs. male)	0.009	0.788
logit(κ)	Country (Glasgow vs. Zambia)	8.891	**<**0.001^1^
	BMI (per unit)	0.284	0.087
	Weight (kg)	0.062	0.378
	Height (cm)	−0.174	0.123
	Age (y)	0.171	0.027^1^
	Sex (female vs. male)	0.478	0.824

1 Statistically significant *P* values at level of significance 0.05.

### Elastic net regression

To confirm the associations identified in the individual linear regression models, we applied ENET to regressions of each parameter against all anthropometric variables together, as well as country and covariate × country interactions, using optimal $\lambda_{1}$ and $\lambda_{2}$ values, corresponding to coefficient shrinkage from ridge regression and lasso regression (Table 4). Using standardized predictors, ENET output for ρ indicated that the country had the greatest impact, and the coefficients of the other predictors and interaction terms were substantially shrunk. (Please note that because of the standardization of the predictors, the coefficients are not directly comparable to TABLE 2, TABLE 3). Applying the ENET model to πρ indicated that weight was a more relevant predictor compared with BMI, whose coefficient was reduced to 0, and suggested that the previous associations between height or sex and πρ were likely confounded by weight. ENET output for logit(κ) shrank the estimate for nearly every predictor to 0, suggesting no association between any anthropometric or demographic predictor and κ (although the effect of country remained).

**TABLE 3:** Regression models for associations between each mechanistic model parameters and each anthropometric and demographic predictor, with a statistical interaction between each predictor and country.

Parameter	Model	Predictors	Model with interaction	
			Coefficient	*P* value
ρ	BMI	BMI (per unit)	0.073	0.310
		Country (Glasgow vs. Zambia)	2.302	0.215
		BMI × country	−0.059	0.452
	Weight	Weight (kg)	0.016	0.371
		Country (Glasgow vs. Zambia)	1.981	0.226
		Weight × country	−0.015	0.539
	Height	Height (cm)	0.018	0.588
		Country (Glasgow vs. Zambia)	9.043	0.207
		Height × country	−0.048	0.257
	Age	Age (y)	−0.096	0.105
		Country (Glasgow vs. Zambia)	−0.792	0.609
		Age × country	0.086	0.159
	Sex	Sex (female vs. male)	0.177	0.724
		Country (Glasgow vs. Zambia)	1.479	0.004
		Sex × country	−0.945	0.177
πρ	BMI	BMI (per unit)	−0.010	0.083
		Country (Glasgow vs. Zambia)	−0.143	0.304
		BMI × country	0.004	0.515
	Weight	Weight (kg)	− 0.003	0.050
		Country (Glasgow vs. Zambia)	− 0.067	0.574
		Weight × country	0.000	0.969
	Height	Height (cm)	−0.004	0.148
		Country (Glasgow vs. Zambia)	−1.209	0.039^1^
		Height × country	0.007	0.052
	Age	Age (y)	0.004	0.456
		Country (Glasgow vs. Zambia)	0.098	0.446
		Age × Country	−0.006	0.241
	Sex	Sex (female vs. male)	−0.073	0.064
		Country (Glasgow vs. Zambia)	−0.153	**<**0.001^1^
		Sex × country	0.156	0.006^1^
Logit(κ)	BMI	BMI (per unit)	0.034	0.918
		Country (Glasgow vs. Zambia)	7.273	0.394
		BMI × country	0.057	0.876
	Weight	Weight (kg)	0.010	0.902
		Country (Glasgow vs. Zambia)	5.717	0.443
		Weight × country	0.046	0.671
	Height	Height (cm)	0.017	0.907
		Country (Glasgow vs. Zambia)	5.474	0.869
		Height × country	0.022	0.912
	Age	Age (y)	0.000	0.998
		Country (Glasgow vs. Zambia)	12.20	0.099
		Age × country	−0.082	0.774
	Sex	Sex (female vs. male)	0.372	0.875
		Country (Glasgow vs. Zambia)	8.814	**<**0.001^1^
		Sex × country	0.160	0.961

1 Statistically significant *P* values at level of significance 0.05.

**TABLE 4:** Elastic net (ENET) regressions standardized coefficient estimates by predictor for each model parameter, ρ, πρ, and logit(κ).

Predictors	Standardized coefficients		
	ρ	πρ	Logit(κ)
(Intercept)	4.192	0.242	8.550
Country	−2.449	0.111	−6.451
Sex	−1.125	0.001	**.**
Weight	−0.207	−0.038	**.**
Height	**.**	0.009	**.**
Age	−0.300	−0.027	**.**
BMI	−0.025	**.**	0.277
Sex × country	1.118	−0.046	**.**
BMI × country	1.080	−0.002	**.**
Weight × country	−0.086	**.**	**.**
Height × country	0.137	0.001	**.**
Age × country	−1.116	0.093	**.**

Predictors include country, sex, weight, height, age, BMI, and interaction terms of each other variable with country.

Predictors for which the ENET model reduced the coefficients to zero are indicated by a (.).

## Discussion

In this analysis, we investigated associations between ^13^C-SBT model parameters and anthropometric variables in adult participants in the United Kingdom and Zambia. Our aim was to better understand how individual anthropometry and demographic characteristics may influence ^13^C-SBT results and to provide insights into the applicability of the ^13^C-SBT in EED research and testing across diverse populations. We identified several statistically significant differences between participants from the United Kingdom and Zambia in the model parameters ρ, which is connected to sucrase-isomaltase activity and liver metabolism; πρ, which is related to an unidentified metabolic process, potentially plasma bicarbonate kinetics, that impacts the breath curve downslope [20]; and κ, the fraction of the tracer recovered on the breath. Notably, Zambian participants exhibited higher values of ρ and κ compared with United Kingdom participants (greater sucrase-isomaltase activity or liver metabolism and higher fraction of tracer recovered), whereas πρ values were on average lower in Zambian participants (slower decay of breath curve). Additionally, body weight was statistically significantly associated with parameter πρ (πρ decreases with weight, resulting in a shallower downslope), which offers clues to understanding which metabolic process it is associated with. This exploratory analysis highlights the utility of our mathematical model for disentangling the different metabolic processes that impact the ^13^C-SBT.

We noted similar associations between body weight and the metabolic rate parameter πρ in both populations. There were differences in dosing between the United Kingdom and Zambia studies, with the latter dosing based on weight (0.3 mg/kg), whereas the former using equal dosing (50 mg) for all participants, regardless of body mass. If both studies had dosed based on body mass, we would not have been able to determine whether the observed association with weight was related to body mass or whether it was a dose effect. However, because weight had a similar magnitude of association with πρ in both countries, it is likely that the true association was with weight or a close anthropometric proxy. Because weight is known to be associated with respiratory rate [21,22], this association may suggest that the metabolic rate controlled by πρ may be related to the rate of plasma bicarbonate exhaled as carbon dioxide [20]. One limitation of the comparison between these studies is that the doses given in the Zambia study were lower overall (∼20 mg at average weight, compared with 50 mg in the United Kingdom study); however, the difference was small—negligible relative to a typical dietary dose—which prior work has demonstrated should have negligible impact on test dynamics. Although risk of unadjusted confounding cannot be entirely excluded, the counterbalancing benefit is that comparing 2 different study populations, with varying risks of EED, reinforces the importance of considering anthropometry in the interpretation of ^13^C-SBT outcomes in future work and highlights the potential implications for understanding and accounting for differences in body composition and metabolic processes across populations.

The association between weight and πρ likely explains the significant effect measure modification observed between sex and country and height and country for πρ. These interactions were likely a result of different relationships between weight and each of sex and height in the 2 countries (Supplemental Table 1). There was a positive relationship between height and weight in United Kingdom participants, but an inverse relationship between weight and height in the Zambia population (albeit statistically nonsignificant). Similarly, males tended to be heavier in the United Kingdom population, whereas females tended to be heavier in the Zambian population. Unfortunately, we had no measures of adipose tissue distribution, for example, waist-to-hip ratio as a measure of visceral compared with subcutaneous fat, which can affect metabolic rates [23]. The results of our ENET regression similarly suggest that weight was the driver of the associations between the anthropometry and πρ, and further emphasized the need to account for potentially different associations between anthropometry and ^13^C-SBT breath curves in different populations.

The Zambian participants had a greater cumulative percent dose recovered at 90 min compared with the United Kingdom participants, despite the much greater likelihood for EED in the Zambian participants. We similarly found that the Zambian cohort had higher ρ values than the United Kingdom cohort, indicating a faster rate of sucrose hydrolysis or liver metabolism. This result is counter to the initial expectation that a population at high risk of EED would have reduced sucrase activity [7,8]. Hence, this result raises questions about the potential influence of diet or other factors on intestinal sucrase activity. In Zambia, the proportion of dietary energy from starchy staples is notably high, where cereals—predominantly maize—continue to comprise the majority of caloric intake [24]. In the United Kingdom, the percentage of calories from protein and fats is proportionally greater than starches, although the free sugar intake is also high [25]. We speculate that these differences in macronutrient composition of the diet may lead to changes in the metabolic function of the intestinal tract. In particular, the small intestine responds to repeated sugar intake by upregulating the expression of sugar transporters and catabolic enzymes [26]. We speculate that the Zambian cohort may have increased capacity to metabolize sucrose and thus have higher values of ρ, the parameter associated with sucrase-isomaltase activity.

The values for parameter κ are clustered near 1 for the Zambian cohort, which suggests that nearly 100% of the dose would be recovered from the breath (as opposed to excreted in urine or sequestered). However, there is also a strong potential that this clustering is an artifact of misspecification of $V_{{CO}_{2}}$, the rate of CO_2_ production, in the Zambian cohort. The calculation of PDRr from the raw isotopic analysis requires an individual-level estimate of $V_{{CO}_{2}}$ [15]. Standard practice is to calculate an approximation of $V_{{CO}_{2}}$ based on estimated body surface area (a function of height, weight, age, and sex). If the value of $V_{{CO}_{2}}$ is not correct, PDRr will be off by a scaling factor. If the $V_{{CO}_{2}}$ estimates were systematically biased for the Zambian population because of, for example, differences in body composition, it would explain the difference in κ between the populations and why the Zambian values were clustered around a less biologically plausible value. Fortunately, the mechanistic modeling approach allows us to compartmentalize the bias in each parameter, so that a potential bias in $V_{{CO}_{2}}$ calculations in Zambian does not invalidate our results for ρ or πρ. However, it does call into question the reliability (and cross-population comparability) of standard summary metrics, ^13^C-SBT and other breath tests; summary metrics like CPDR90 are highly sensitive to misspecification of the scale of PDRr. Future studies should consider the validation of $V_{{CO}_{2}}$ calculations through indirect calorimetry.

In addition to the limitations mentioned above, although the Zambian participants were drawn from a population considered to be at high risk of EED, the previous analysis of the Zambian cohort found no significant correlations between intestinal biopsy morphometry or sucrase-isomaltase activity and breath test measures [16]. Accordingly, we cannot determine whether differences between the United Kingdom and Zambian cohorts reflect differences between individuals with versus without EED. Additionally, the differences in BMI and age group between the United Kingdom and Zambia cohorts may have induced selection bias and affected the generalizability of findings; further analysis of confounding is needed in larger studies. Finally, both study cohorts were relatively small, so all associations found here should be considered preliminary.

Our study has several strengths that enhance the reliability and validity of our findings. First, our application of the mechanistic model allowed us to consider the different metabolic processes separately and to isolate associations between these processes and anthropometry. By using ENET to control for confounding associations between variables, we were able to better isolate the effect of specific variables on the ^13^C-SBT parameters. Finally, by comparing adult populations in both the United Kingdom and Zambia, we provided insights into the influence of differing physiological factors on intestinal function and sucrase activity. Our results highlight the importance of considering population-specific factors in future ^13^C-SBT studies.

Our study provides valuable insights into the associations between ^13^C-SBT parameters and anthropometric variables, drawing attention to the interaction of physiological factors in metabolic function and sucrase-isomaltase activity. Our findings highlight the need for further research to explore the true impact of EED on small intestine pathophysiology and to validate the utility of the ^13^C-SBT as a diagnostic tool across heterogeneous populations. By addressing these gaps in research, we can advance our understanding of the ^13^C-SBT’s connection with gut health and contribute to the development of targeted diagnostic tools to improve our understanding of gastrointestinal disorders such as EED globally.

## Author contributions

The authors’ responsibilities were as follows – DJM, PK, CAE: designed the original research studies; AFB, GOL: designed the secondary analysis research study; RJS, CAE, PH, EB, PK, DJM: conducted the research; SPI, HVW: analyzed the data; SPI, AFB: wrote the article; AFB: had primary responsibility for final content; and all authors: read and approved the final manuscript.

## Data availability

Data described in the manuscript, code book, and analytic code will not be made available because they contain sensitive personal information

## Funding

This project was funded through the International Atomic Energy Agency (IAEA) coordinated research projects E4.10.16 and E43036, United States National Science Foundation (NSF) grant DMS1853032, and United States National Institutes of Health (NIH) grant K01AI145080. The NSF and NIH were not involved in study design; collection, analysis, and interpretation of data; writing of the report. The IAEA was involved in study design of the data collection. We also acknowledge additional funding from Nutriset, France that enabled ^13^C-SBT data collection in the UK. Nutriset was not involved in the study design; collection, analysis, and interpretation of data; or writing of the report.

## Conflict of interest

DJM reports financial support was provided by International Atomic Energy Agency. PK reports financial support was provided by International Atomic Energy Agency. GOL reports financial support was provided by International Atomic Energy Agency. AFB reports financial support was provided by National Science Foundation. GOL reports financial support was provided by National Institutes of Health. DJM reports financial support was provided by Nutriset SAS. If there are other authors, they declare that they have no known competing financial interests or personal relationships that could have appeared to influence the work reported in this article.
