# Supplementary material for: Associations of 13C-Sucrose Breath Test Dynamics with Anthropometry and Demographics: A Comparison of Studies in the United Kingdom and Zambia
Source: Curr Dev Nutr. 2025 Oct 30;9(11):107590. doi: 10.1016/j.cdnut.2025.107590 (PMC12666799; doi:10.1016/j.cdnut.2025.107590)
Supplement: multimedia component 1 [file mmc1.docx]

Supplemental material for Iorga et al., *Associations of ^13^C-sucrose breath test dynamics with anthropometry and demographics: a comparison of studies in the United Kingdom and Zambia*.

**
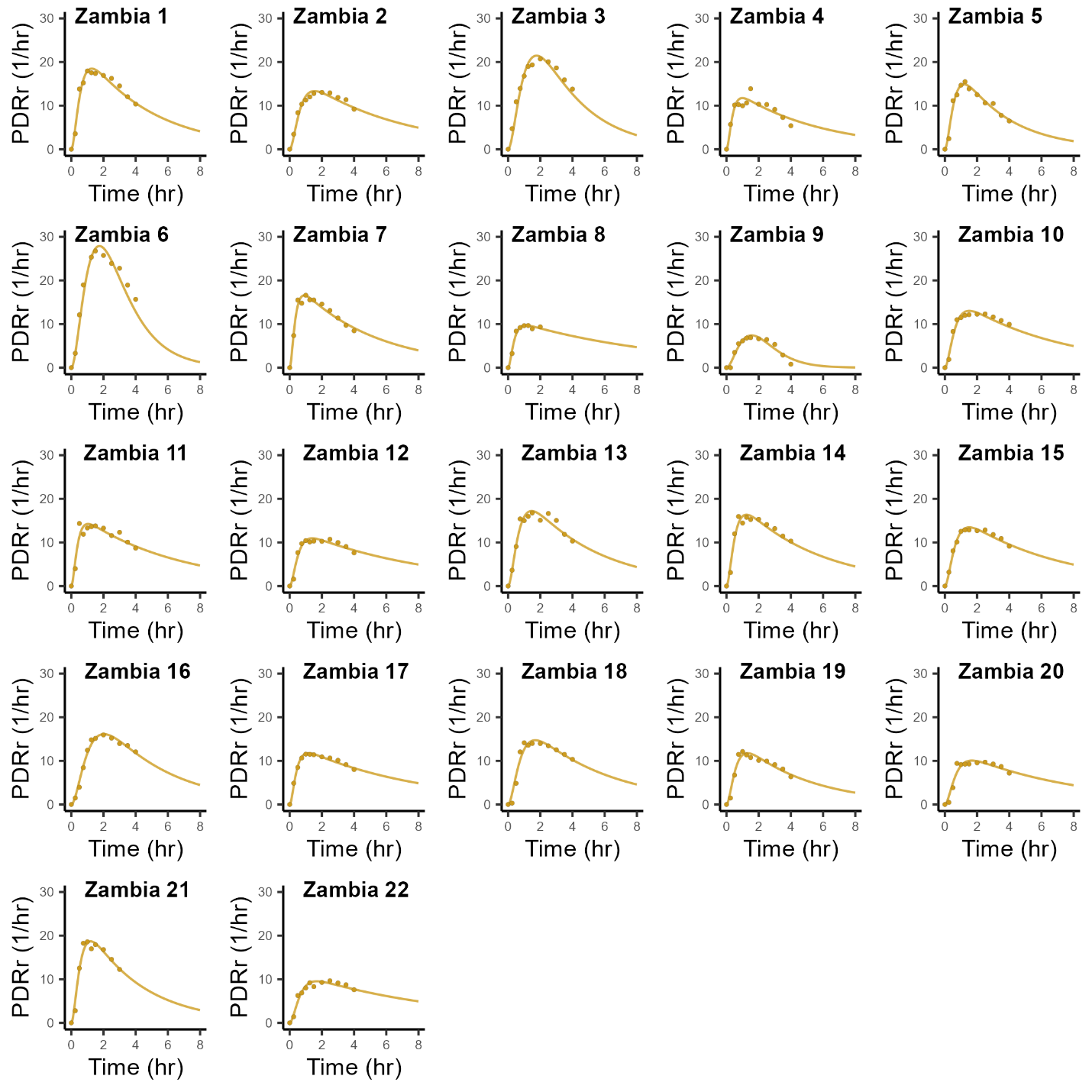
**

**Supplemental Figure 1**. ^13^C-Sucrose breath test trajectories for 22 Zambian participants with percent dose recovery rate (PDRr) measurements (points) overlaid with their corresponding best-fit mechanistic model curve (lines). The y-axis is consistent between plots to allow for better visualization of varying peak heights. The x-axis is extended to 8 hours for Zambian participants despite only collecting up to 4 hours of data to between-study comparison of the data and curves.


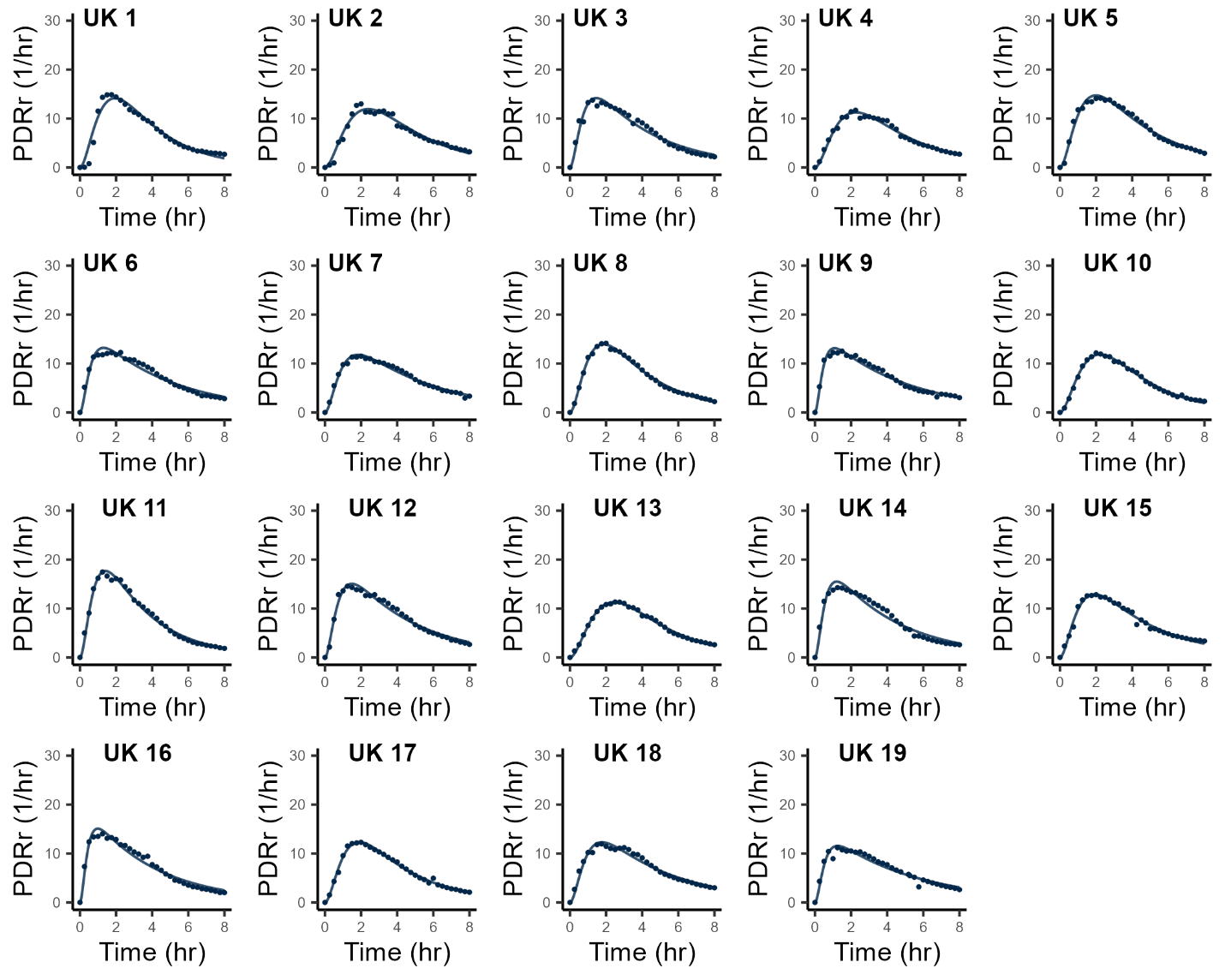


**Supplemental Figure 2**. ^13^C-Sucrose breath test trajectories for 19 United Kingdom (UK) participants with percent dose recovery rate (PDRr) measurements (points) overlaid with their corresponding best-fit mechanistic model curve (lines). The y-axis is consistent between plots to allow for better visualization of varying peak heights.

**Supplemental Table 1:** Exposure–covariate table assessing the potential for confounding the relationship between each predictor and the outcomes by weight for each country. Mean values are given for weight, age, height and BMI, while proportion of males is provided for the variable sex. These results suggest that weight has the potential to confound the relationship between the mechanistic parameters and each of age, sex, height, and BMI.

|  |  | Quintiles of weight | | | | |
| --- | --- | --- | --- | --- | --- | --- |
|  |  | Quintile 1 | Quintile 2 | Quintile 3 | Quintile 4 | Quintile 5 |
| UK | Weight (kg) | 49.4 | 58.2 | 65.0 | 71.6 | 90.1 |
|  | Age (years) | 20.5 | 25.8 | 20.8 | 21.5 | 27.3 |
|  | Sex (% Male) | 0% | 25% | 75% | 50% | 100% |
|  | Height (cm) | 165 | 167 | 173 | 175 | 180 |
|  | BMI | 18.2 | 21.1 | 21.6 | 23.3 | 27.8 |
| Zambia | Weight (kg) | 53.5 | 59.2 | 65.4 | 71.8 | 95.0 |
|  | Age (years) | 46.0 | 39.5 | 29.2 | 47.5 | 38.0 |
|  | Sex (% Male) | 60% | 100% | 75% | 0% | 0% |
|  | Height (cm) | 162 | 175 | 166 | 159 | 157 |
|  | BMI | 20.5 | 19.3 | 23.8 | 28.5 | 38.7 |

**Supplemental Table 2:** Exposure–covariate table assessing the potential for confounding the relationship between each predictor and the outcomes by age for each country. Mean values are given for weight, age, height and BMI, while proportion of males is provided for the variable sex. These results suggest that age has the potential to confound the relationship between the mechanistic parameters and each of sex, weight, height, and BMI.

|  |  | Quintiles of Age | | | | |
| --- | --- | --- | --- | --- | --- | --- |
|  |  | Quintile 1 | Quintile 2 | Quintile 3 | Quintile 4 | Quintile 5 |
| UK | Age (years) | 19.2 | 20.0 | 21.8 | 24.8 | 31.0 |
|  | Weight (kg) | 61.9 | 62.7 | 62.4 | 76.7 | 64.1 |
|  | Sex (% Male) | 25% | 50% | 50% | 75% | 33% |
|  | Height (cm) | 170 | 172 | 171 | 175 | 169 |
|  | BMI | 21.6 | 21.1 | 20.9 | 24.8 | 22.3 |
| Zambia | Age (years) | 22.0 | 32.0 | 44.2 | 51.2 | 56.8 |
|  | Weight (kg) | 65.4 | 69.4 | 70.9 | 76.2 | 60.0 |
|  | Sex (% Male) | 80 | 50 | 25 | 25 | 50 |
|  | Height (cm) | 166 | 164 | 162 | 162 | 164 |
|  | BMI | 24.3 | 26.4 | 27.5 | 29.3 | 22.6 |
